# Equine Crofton Weed (*Ageratina* spp.) Pneumotoxicity: What Do We Know and What Do We Need to Know?

**DOI:** 10.3390/ani13132082

**Published:** 2023-06-23

**Authors:** Frances Marie Shapter, José Luis Granados-Soler, Allison J. Stewart, Francois Rene Bertin, Rachel Allavena

**Affiliations:** School of Veterinary Science, University of Queensland Gatton, 5391 Warrego Highway, Gatton, QLD 4343, Australia; j.granados@uq.edu.au (J.L.G.-S.); allison.stewart@uq.edu.au (A.J.S.); f.bertin@uq.edu.au (F.R.B.); r.allavena@uq.edu.au (R.A.)

**Keywords:** horse, lung, fibrosis, pneumonia, toxic plant, pulmonary fibrosis, dyspnea, *Ageratina*, *Eupatorium*

## Abstract

**Simple Summary:**

Crofton weed toxicity, caused by *Ageratina adenophora*, has been recognized as a cause of fatal lung disease in horses for over a century. Despite its impact on horse health in many areas of the world, the toxic syndrome is poorly understood and understudied. This paper looks at the prior research on weed biology, the potential toxicology mechanisms, and the pathology in horses and other species, as well as the future directions to improve our understanding of this fatal toxic weed affecting horses.

**Abstract:**

Crofton weed (*Ageratina adenophora*) is a global and highly invasive weed, with ingestion causing severe respiratory disease in horses, leading to irreversible and untreatable pulmonary fibrosis and oedema. While reports of equine pneumotoxicity remain common in Australia and New Zealand, equine pneumotoxicity may be underdiagnosed in other countries where Crofton weed is endemic but poorly differentiated. The pathogenesis of Crofton weed toxicity following ingestion has been well described in a number of different animal models, including rodents, rabbits, and goats. However, induced toxicity is organ-selective across different animal species, and these vastly differ from the pathogenesis described in horses, both clinically and after experimental exposure. Sources of variation may include species-specific susceptibility to different toxins present in the plant, different mechanistic processes of toxicity, and species differences in toxin biotransformation and bioactivation across different organs. Considering disease severity and Crofton weed’s invasiveness globally, assessing published toxicological and exposure data is necessary to advance research, identify specific toxins for horses, and possible prophylactic and therapeutic strategies. This review presents an overview of the available literature on equine toxicity, parallels between toxicity in horses and other animal species, and important aspects to be included in the future research agenda.

## 1. Introduction

It has been a century since Crofton weed (*Ageratina adenophora*, syn: *Eupatorium adenophora*, *E. glandulosum*) was identified as a plant of interest in relation to horse deaths due to pulmonary disease [1,2]. Despite this, little is known about the pathophysiological mechanisms that result in pulmonary fibrosis and the reasons why horses appear to be more susceptible than other species. Crofton weed is native to Central America but is now present in many tropical and subtropical countries as a highly invasive noxious weed, whose uncontrollable spread is characterized by allelochemical competition and alterations of soil microbial communities [3,4]. Although Crofton weed is well known for its negative impacts on ecology, agriculture, and animal health [4,5,6,7], it has diverse medicinal properties and has been used in traditional medicines and ethno-pharmacology. Some of the 34 phytochemicals and 52 volatile oils that have been isolated are currently under investigation for potential pharmacological uses [8,9,10,11,12,13,14,15].

There are no confirmative diagnostic tests or efficacious treatments for Crofton weed-associated pneumotoxicity in horses. A definitive diagnosis of the disease is not possible, and a diagnosis can be formulated only presumptively, based on the history of exposure and exclusion of diseases with similar clinicopathological findings. Crofton weed intoxication in horses causes severe respiratory disease, with clinical signs including coughing, increasingly severe exercise intolerance, tachypnoea, and adventitial respiratory sounds. [1,16,17]. The disease progresses to weight loss, respiratory distress, and cyanosis preceding death, attributed to pulmonary fibrosis, intense pulmonary oedema, and biventricular cardiac failure following hypoxia-associated cor pulmonale [1,16,17]. Histopathologically, Crofton weed induces a chronic multinodular pulmonary fibrosis with interstitial pneumonia and pulmonary oedema [1,16,17]. As terminal cases are usually diagnosed late in the disease process, Crofton weed toxicity is considered untreatable and irreversible, and euthanasia remains the most humane endpoint for affected horses. Antemortem pulmonary biopsy with histopathology will rule out other differentials such as EHV5-associated equine pulmonary multinodular fibrosis and fungal infection (such as *Pneumocystis carinii*) supported by PCR and serology, and could be used to produce a provisional diagnosis earlier in the disease process [18,19,20,21,22,23].

Despite confirmation of Crofton weed as the cause of fatal pneumotoxicity in horses [16,17], the phytotoxins involved and pathophysiological mechanisms remain unclear. Equine Crofton weed pneumotoxicity—also known as “Crofton Weed Poisoning”, “Numinbah Horse Sickness”, and “Tallebudgera Disease”—was originally attributed to a possible allergic response or to the hematogenous distribution of an unknown phytotoxin after ingestion [16,17]. In contrast to horses, Crofton weed ingestion is predominantly linked to hepatotoxicity in other animal species, which has been associated with terpenoids [16,24,25,26,27,28,29]. As different organs are affected across several animal species, specific target organ phytotoxins, as well as different routes of exposure, have been proposed [7,14,25,26,30]. However, the possible role of species-specific metabolic bioactivation of Crofton weed toxins as a basis for organ-selective toxicity has not been investigated, but remains plausible.

Equine Crofton weed toxicity research ceased in the 1980s, but other disciplines have extensively investigated Crofton weed biology, control, phytochemical properties, potential for pharmaceutical development, and toxicity in different animal species [3,4,14]. This review presents an overview of the available literature on Crofton weed toxicity in horses, toxicity in other animal species and parallels with the equine disease, and possible prophylactic and therapeutic strategies.

## 2. Crofton Weed Identification, Morphology, and Impact

### 2.1. Identification of Crofton Weed

Field identification is paramount, as currently, prevention is the only cure. Once identified within paddocks, horse owners should be advised to prevent any grazing of infiltrated pastures by fencing off infested areas, and rehabilitating quarantined paddocks with the use of approved herbicides, mechanical control, and planting competitive replacement pastures [31,32]. In China and India, the invasive nature of this species has threatened native biodiversity and resulted in significant research into control strategies [3,5,33,34,35]. Despite successful biological control documented in Hawaii during the 1920s, the biological control strategies applied in Australia were not successful [31,32,36,37,38]. Once the plant is established in grazing areas, physical and chemical control have limited effectiveness considering rapid seed dispersal, plant regrowth, and negative impacts of pesticides on native flora and fauna [31,32,38]. In addition to the cited literature, there are many online resources that have weed control advice, specific to each region, which are being constantly updated as new chemical and rehabilitation protocols emerge. Owners should be advised to seek the latest, local information for both *Ageratina* spp. control methods.

**Figure 1:**
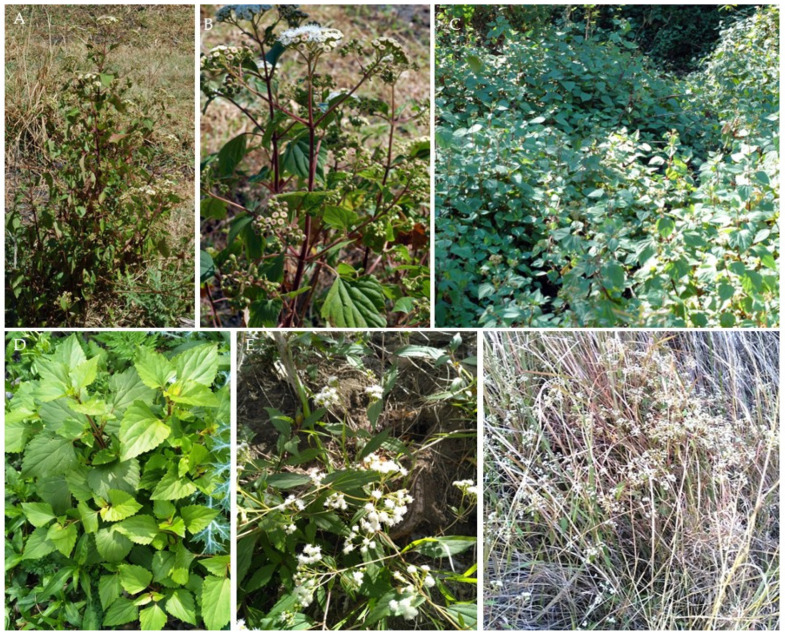
Examples of different life stages and growth habits of *A. Adenophora* (**A**–**D**) and *A. riparia* (**E**,**F**). Plates (**A**,**B**) show the erect burgundy stems, with opposite arrangements of bright to dark green leaves. Plates (**C**,**D**) show the rhomboid or trowel-shaped leaves with serrated edges and demonstrate its prolific growth creating a continuous hedge of *A. Adenophora*. Plates (**E**,**F**) demonstrate the spear-shaped leaves with toothed edges and a pointed tip, prostrate growth habit, and reduced biomass of *A. riparia*. Plates (**B**,**E**) show the similarity between the flower morphologies of both species.

Also known as Eupatory, Sticky Snakeroot, Catweed, Banmara, Maui Pakami, Sándara, Flor de Espuma, and Mexican Devil, *A. adenophora* is a member of the Asteraceae family. The plant is a perennial large herb or under-shrub with multiple erect glandular and hairy burgundy stems reaching a height of 1–2 m (Figure 1A,B). Its leaves grow in an opposite arrangement, are 2.5 to 5 cm long, and are dark green, broad, trowel-shaped with serrated margins, and burgundy petioles (Figure 1C,D). The foliage is distinctly and highly aromatic when disturbed or crushed. Flowers are 5–8 mm wide and grow in white clusters of disc florets, appearing as small, dense heads at the ends of branches during spring. Small leaf-like structures called bracts surround the flowers. Although Crofton weed is apomictic (able to produce female clones from asexual seed formation) it is a prolific pollen producer. A mature plant can produce 100,000 to 1,000,000 seeds per year, which are very light (25,000 seeds/g), slender, angular, 2 mm long, and almost black, with fine white hairs at the tip [31,32,35,39]. Crofton weed and the less prolific Mist weed (*Ageratina riparia*, syn: *Eupatorium riparia*), which is commonly found along waterways, are sometimes mistaken for each other due to their flowers being almost identical. However, when it is not flowering, Mist weed is often not recognized at all, as it is far less visible than the erect Crofton weed, but evidence indicates that both are toxic when consumed [17]. While the flowers and odor of *A. riparia* are very similar to those of *A. adenophora*, *A. riparia* stems can be pale green rather than burgundy, have a short (<50 cm height) prostrate habit, and have leaves with an elliptic shape. (Figure 1E,F) [32].

### 2.2. Pollen Morphology and Investigation as a Potential Alveolar Mechanical Irritant

**Figure 2:**
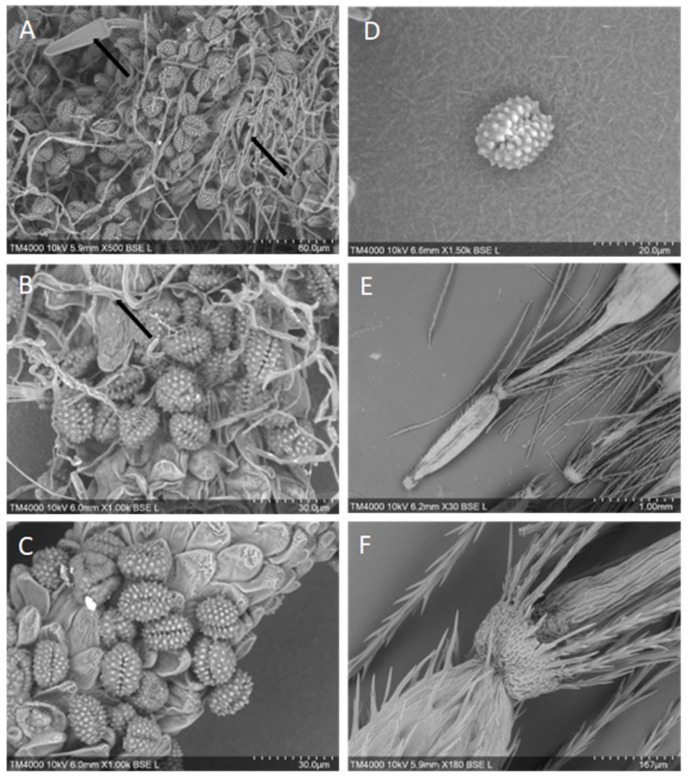
Pollen and achene morphology of Crofton weed, *A. adenophora* (**A**,**B**), and Mist weed, *A. riparia* (**C**–**F**), using scanning electron microscopy. Plates (**A**,**B**) Note the presence of fungal fruiting bodies and hyphae intertwined amongst the pollen granules, as indicated by arrows. Plates (**C**,**D**) note pollen morphology are very similar to that of other *Ageratina* spp. [40]. Plates (**E**,**F**) barbed achenes.

Many of the plants of the family Eupatorium have had their pollen morphology characterized, with 11 of the 192 species exhibiting short-spined pollen typical of plants associated with allergic responses [40,41]. However, the pollen from *A. adenophora* and *A. riparia* has not been characterized by a Scanning Electron Microscopy (SEM). The Environmental Analysis Laboratory at Southern Cross University, Lismore, was contracted by the authors to perform SEM of *A. adenophora* and *A. riparia* pollen samples collected from Bentley, Northern NSW. Twelve pollen samples from *A. adenophora* and *A. riparia* were prepared via desiccation, attachment to SEM stubs, and examined under a low vacuum. A Hitachi TM4000 desktop SEM was used to evaluate and record the images. No differences between the Crofton weed and the Mist weed pollen were noted, with both being spherical to ovoid and with one colpi. Pollen ranged from 15–22 µm in size, with continuous spikes across the surface (Figure 2A–D). The barbellate achenes (seed capsules: ~1–2 mm) had sharp barbs across the surface of the seed and papillae (Figure 2E,F), and all the samples contained fungal hyphae that were intertwined with the reproductive structures (Figure 2A,B).

Due to the predominant timing of the clinical signs of equine pneumotoxicity falling 6–10 weeks after the onset of the flowering season [11], combined with the high volume of flowers, seeds, and pollen produced, the role of the floral components and or pollen inhalation in pneumotoxicity has been considered. In 1958, one report from China attributed the death of 200 horses as a result of asthma brought on by pollen from Crofton weed flowers [42]. However, as Crofton weed pollen is greater than 5 µm in size, it is too large to enter the alveoli, and therefore, a direct mechanical cause of the pulmonary damage in horses is considered unlikely. Additionally, feeding trials using non-flowering plants and intragastric tubes feeding across different species have demonstrated that pollen inhalation is not necessary to elicit toxicity [16,17,43,44].

### 2.3. Geographic Distribution of Crofton Weed Toxicity in Horses

During the 1920s “Blowing disease” was anecdotally associated with Maui Pamakani, the local Hawaiian common name for Crofton weed introduced in about 1860 in the Maui Island [1,2,36] The etiology of the described disease in Hawaii was never confirmed, as feeding and pollen inhalation trials were negative; however, the descriptions were identical with the first reports of Numinbah Horse Sickness in Australia during the 1950s [1]. Pamakani was postulated to cause the death of a large number of horses pasturing above certain altitudes (600 m above sea level) in Maui [1,2]. However, despite its presence, it was reported to cause no injuries to horses on Hawaiian islands other than Maui [2]. Interestingly, reports declined during weeding periods, and apparently, they ceased after the establishment of biocontrol by a stem gall fly (*Procecidochares utilis*) in 1945 [1,36,37,45,46].

In Australia, the plant escaped domestication in Sydney in 1904, and by the 1940s, it had established itself as a weed throughout coastal New South Wales (NSW) and Southeast Queensland. Accordingly, the first reports of the disease in Australia were documented in 1941 in the extreme northern part of NSW and southern Queensland [1]. Preceding World War II, Crofton weed toxicity was commonly suspected in Australian Draught Horses working in the valleys and mountains of south-eastern Queensland and north-eastern NSW, and from the year 1948, reports continued to increase [1,16]. In 1952, *P. utilis* was unsuccessfully introduced as Crofton weed biocontrol in Australia, and by 1954, the NSW Institute of Inspectors of Stock yearbook was reporting outbreaks of “Numinbah Horse Sickness”, estimating that during the decade preceding the report, hundreds of horses had died from this disease [1,25,37]. By the 1970s, respiratory problems increased in regions where Crofton weed was common [16]. Farms infested with the plant in Australia sporadically reported cases of suspected Crofton weed pneumotoxicosis in the summer, affecting horses of all ages and occurring a minimum of two months after plant ingestion in the spring [47].

As a confirmative diagnosis is not possible, the actual prevalence and worldwide distribution of Crofton weed intoxication in horses are largely unknown. Outside Australia, it has been suggested that the disease is also present in New Zealand, possibly China, and the Himalayas [25,47,48]. However, despite the wide distribution and reported invasiveness of Crofton weed in Burma, Fiji, India, Jamaica, Malaysia, Nepal, Pakistan, Singapore, South Africa, Spain, Sri Lanka, Thailand, the Philippines, the Pacific Islands, the United States, and Vietnam, pneumotoxicity in horses is not commonly observed or officially documented, and reports are mostly anecdotal [4,15,32,39,49,50,51]. However, in some of these countries, local differences in horse husbandry and population size might also lead to reduced exposure, even when the plant is present.

Despite the wide distribution and invasiveness of Crofton weed [4,15,32,39,49,50,51], pneumotoxicity in horses seems to be limited to some specific locations, and to our knowledge, it has not been reported in countries where the plant is native. This may be explained by specific factors impacting the concentration of phytotoxins that are present in the plant across different geographic locations. For instance, climate, soil, and other environmental variables influence the synthesis and content of phytochemicals, which are known to vary with geographical region in several plant species [52,53]. Indeed, the allelopathic activity of volatile organic compounds and the foliar concentration of various terpenes, which are part of the defensive arsenal of *A. adenophora*, are different between plants from native and non-native locations [54,55]. A comparative assessment of phytotoxins in plants collected from regions where the disease is present and absent is necessary to compare the concentrations of specific toxins and identify the reasons why horses might be at a higher risk of developing toxicity in certain geographic areas.

## 3. Phytotoxicity

### 3.1. Crofton Weed Phytochemical Analysis

Crofton weed is rich in bioactive phytochemicals such as benzofuran, coumarins, flavonoids, phenolic acids, phenylpropanoids, polysaccharides, quinic acid, chromene derivatives, sterols, alkaloids, and mono-, sesqui-, di-, and tri-terpenoids [5,14,15,56,57,58]. A 2020 review identified 34 phytochemicals and 52 volatile oils that were isolated from Crofton weed [14]. There is a plethora of research investigating pharmacological applications for Crofton weed’s chemical extracts and phytotoxins, including its antimicrobial, anti-inflammatory, anti-pyretic, wound-healing, anti-oxidant, analgesic, anti-tumor, anti-viral, insecticidal, larvicidal, and acaricidal activities [14,59,60,61]. Additionally, differential production and activity of allelopathic phytochemicals between plant specimens from native and non-native regions have been documented [54,55]. Pyrrolizidine alkaloids may potentially play a role in Crofton weed toxicity in horses [47]. Food safety concerns resulting from pyrrolizidine alkaloid contamination during the production of honey and pollen products have facilitated significant research confirming the presence of these phytochemicals in the pollen of many plants in *Ageratina* genus [15,62,63,64,65]. Although one phytochemical study reported the isolation of alkaloids from Crofton weed leaves, the type of alkaloid detected in that study was not further characterized [57].

### 3.2. Feeding Trials in Horses

The results of feeding trials were congruent with the current and historical reports from horse owners and veterinarians that disease outbreaks tend to occur in late spring through summer, corresponding to the time coincident with and after Crofton weed’s annual flowering season. The first feeding trial confirming the suspected etiology of Crofton weed toxicity in horses was published in 1979 [16]. Two horses were fed whole plant materials, including the flowering heads (Table 1). One horse was fed Crofton weed over eight months and developed the clinical signs and severe pathological pulmonary changes that are typically observed in field cases [16]. The second horse was fed the plant over 42 days, and although no clinical abnormalities were observed, less severe pulmonary pathological changes were also documented at necropsy [16]. The findings of this first trial supported a possible allergic reaction, and although no microbiological growth was detected from the pulmonary samples, a secondary infectious process was suspected, and the negative cultures were attributed to antibiotic treatment that was administered prior to euthanasia [16]. Because the horses were fed in deep troughs, which might have facilitated the inhalation of all small particles including pollen, pneumotoxicity following pollen inhalation was not ruled out [16].

In a subsequent study published by the same research group, the toxicity of the closely related plant Mist weed (*A. riparia*) was determined utilizing two horses fed plant material that was picked during its flowering season [66]. Mist weed induced clinical signs and necropsy findings similar to Crofton weed, suggesting a common phytotoxic mechanism [66]. The two species also share a common geographic distribution and some habitats, though Crofton weed is far more prevalent due to its ability to colonize open, moist regions, while Mist weed rarely colonizes beyond a riparian zone [33,34]. Furthermore, Crofton weed is an erect, multi-stemmed shrub that forms dense, tangled bushes of up to 2.0 m height, producing large quantities of biomass compared to the comparatively low biomass of the prostrate Mist weed [32,33,34]. While this trial confirmed Mist weed’s toxicity in horses, there have been no case reports of spontaneous disease linked to Mist weed, and this may be due to a lack of readily ingestible volumes or its co-habitation with the more abundant Crofton weed.

A second Crofton weed feeding trial hypothesized that the disease was caused by either the (1) ingestion of flowers, (2) inhalation of pollen, or (3) an increased concentration of phytotoxins in the foliage during plant flowering [17]. To test these hypotheses, ten horses were fed 3–4 kg of Crofton weed daily. Two horses were fed the flowering plant only (for 50 and 90 days, one by consumption and the other by the administration of blended plant materials via the stomach tube, respectively). Four horses received non-flowering plants only (for 93, 164,164, and 327 days), and another four horses were fed both flowering and non-flowering plants (for 165, 415, 442, and 442 days) [17]. While the sample size and variability in days of feeding excluded the use of statistical analysis, the data generally supported several key assumptions, which are as follows: (1) non-flowering plants were also toxic, though at a reduced level than flowering plants; (2) necropsy findings were more severe with prolonged feeding times and when the diet included flowering plants; (3) early lung lesions in the absence of clinical signs were detected after only 57 days in horses that were fed flowering plants; and (4) once the pulmonary lesions occurred, the damage appeared to be irreversible and cumulative with re-exposure [17]. Based on the single horse fed by stomach tube having widely distributed focal pulmonary lesions after 90 days and the results from the necropsies performed on all ten horses, it was concluded that hematogenous dissemination of an unknown toxin was more likely than inhalation [17]. No further equine feeding trial expanded on these conclusions, and it is unknown if any signs of inflammation occur in peripheral blood with Crofton weed toxicity, which may confuse its antemortem diagnosis with other infectious causes of pulmonary oedema and fibrosis in horses. Additional research using different animal models has continued to explore Crofton weed toxicity, the involved phytotoxins, and the mechanisms of toxicity (Table 1).

### 3.3. Postmortem Findings of Equine Crofton Weed Toxicity

Crofton weed induces interstitial pneumonia characterized by multinodular pulmonary fibrosis in horses. The necropsy findings from early feeding trials confirmed that chronic lesions developed into extensive and severe fibrosis, which appeared in the gross tissue as white nodular lesions that did not collapse and generalized throughout the lung [16,17]. The normal pulmonary architecture was effaced by fibrous tissue, with few alveoli remaining and less severe areas displaying a proliferation of type II pneumocytes [17]. In the 1979 feeding trial, the disease was suspected to be triggered by an allergic response, due to proteinaceous fluid present in the alveoli and vascular damage resulting from a loss of capillary integrity, progressing to a secondary infection and abscessation [16]. However, the histopathologic findings from the 1985 trial described interstitial pneumonia with type II pneumocyte hyperplasia, clusters of interalveolar macrophages with lymphocytes infiltrating within interlobular septa, and perivascular fascia. In addition to the pulmonary changes, a single study described cardiac dilation with slight hydropericardium, after Crofton weed ingestion in horses, with other abnormalities attributed to heavy parasite infestation [1]

It is now believed that circulating absorbed phytotoxins such as pyrrolizidine alkaloids and ketones originating from the ingestion of Crofton weed could trigger interstitial pneumonia in horses. Other differentials for interstitial pneumonia in horses include lungworm infections, Influenza virus, Hendra virus, and toxins, such as Paraquat and 3-methylindole, while differentials for pulmonary fibrosis include Equine Herpesvirus 5 (EHV 5)-associated equine multinodular pulmonary fibrosis silicosis and fungal pneumonia, such as from *Pneumocystis carinii* [18,19,20,21,22,23,67,68]. Most infectious causes would likely have clinicopathological evidence of inflammation in the peripheral blood such as a combination of some of the following: anemia of chronic infection, hyper or hypo-gammaglobulinemia, hypalbuminemia, hyperfibrinogenemia, low serum iron concentrations, elevated serum amyloid A concentrations, leukocytosis, toxic changes in leukocytes, and possibly the presence of band neutrophils. Although unlikely, it is unknown whether Crofton weed pneumotoxicity causes any changes in serum biochemistry or hematology. Terminally, there would likely be hypoxemia and a respiratory acidosis.

### 3.4. Species Differences in Crofton Weed Organ-Selective Toxicity

Crofton weed induces organ-selective toxicity across different animal species, which is possibly mediated by different phytotoxins or different species-specific metabolic mechanisms. A multispecies feeding trial performed in 1979 included rabbits (*n* = 2), sheep (*n* = 2), and rats (*n* = 4) [16]. Both rabbits were fed Crofton weed for nine months and developed pulmonary microscopic changes similar to early lesions observed in horses, while sheep and rats did not display any pulmonary abnormalities during pathologic examination; however, it is not definitively stated if other organs were examined for microscopic changes [16]. Although that study did not identify any abnormalities in sheep and rats, subsequent trials reported specific target organs across different species (Table 1). Specifically, the oral intake or intragastric administration of Crofton weed or its extracts lead to renal, splenic, and liver toxicity in goats; liver, spleen, and intestinal toxicity in rodents; and negative impacts in the digestive function of cattle (Table 1) [16,17,24,25,27,28,29,44,51,69,70,71,72,73,74,75,76,77].

The phytochemicals 9-oxo-10, 11-dehydroagerophorone (Euptox A), 2-deoxo-2-(acetyloxy)-9-oxo-ageraphorone (DAOA) and 9-oxoagerophorone (OA) have been identified as the major phytotoxins in *A. adenophora*, and have been demonstrated to induce hepatotoxicity in rats and mice [25,43,71,76]. Euptox A hepatotoxicity in rodents has been demonstrated after toxin purification [25,43,56,71,76]. Furthermore, the oral administration of Euptox A was associated not only with hepatotoxicity, but also with spleen and intestinal toxicity in rodents [43,76]. Oxidative stress and inflammation were postulated as the main mechanistic processes leading to animal disease after Crofton weed ingestion [7,26]. Further mechanistic studies in mice revealed that Euptox A induces cell cycle arrest and apoptosis in hepatocytes via the accumulation of reactive oxygen species (ROS) and in splenocytes autophagy by disrupting the p38 MAPK- and PI3K/Akt/mTOR-mediated pathways [43,44]. These findings are like those of feeding trials in goats, demonstrating the induction of cell cycle arrest, apoptosis, and autophagy in renal cells, splenocytes, and hepatocytes, highlighting the possible role of Euptox A and other terpenes behind the observed toxicity in goats [27,28,29]. The role of Euptox A and other terpenes as responsible for the toxic effects of Crofton weed in horses has not been investigated.

Considering the presence of alkaloids in Crofton weed and the reported pneumotoxicity of these phytochemicals in horses after the ingestion of alkaloid-rich plants [19,78], pyrrolizidine alkaloids have also been anecdotally suspected to play a role in equine Crofton weed pneumotoxicity [47]. Similar to Euptox A, pyrrolizidine alkaloids typically induce hepatotoxicity in rodent models [79]. Pharmacokinetics studies of ingested pyrrolizidine alkaloids in mice and rats, demonstrated preferential bioactivation in the liver, resulting in hepatotoxicity and secondary pneumotoxicity after the migration of liver-derived dehydro-pyrrolizidine alkaloids and the intrapulmonary formation of pyrrole–protein adducts [78,79,80,81]. Species differences in the concentration of biotransformative enzymes in the liver and lungs of horses and other animal species may explain the differences in organ toxicity between species [82,83,84,85,86]. As observed with other toxicities in horses, a predominant cytochrome P450-mediated pulmonary bioactivation of phytotoxins in the Clara cells, also known as club cells or bronchiolar exocrine cells, of horses might be a possible driver for Crofton weed-associated pulmonary-selective toxicity [78,85,86,87].

## 4. Discussion

### 4.1. Gaps in Clinical Diagnostics

It is likely that chronic long-term ingestion of Crofton weed would result in a gradual progression of clinical signs in horses. The toxic dose required to induce subtle clinical signs is unknown. Published feed trials that recorded pathological findings utilized generous doses of weed. Changes in hematology and serum biochemistry have not been investigated, but knowledge of any bloodwork changes (or lack thereof) resulting from Crofton weed pneumotoxicity would improve diagnostic capacity. Changes in arterial blood gas analysis (at rest and after controlled exercise), thoracic ultrasound, and radiographs after controlled experimental exposure would be useful to confirm the severity of interstitial pneumonia antemortem. Fluid from a transtracheal wash or bronchoalveolar lavage can be utilized for cytology, microbiological cultures, and PCR and are important in the work-up of clinical cases and samples that should be collected during experimental exposure. Despite some risk of pulmonary hemorrhage, an antemortem lung biopsy can be useful for providing samples for histopathology, cytology, microbiological cultures, and PCR to help determine the presence of etiological agents [18,67,88,89]. Although the biopsy findings would not be pathognomonic for Crofton weed toxicity, the documentation of the progressive disease after experimental exposure would be useful when paired with other clinical parameters. In clinical cases where Crofton weed exposure has occurred, histopathology could indicate a decreased likelihood of other differentials, which may have a better prognosis with an earlier, specific therapy.

### 4.2. Areas Requiring Further Research

Crofton weed pneumotoxicity in horses has been known for over a century. Although it is a recognized differential for interstitial pneumonia, no case series has been published [20,22]. It is unknown whether the removal of subclinically affected horses from contaminated pastures would lead to a possible cessation of disease progression. Additional therapeutic strategies have not been investigated. Although experimental disease induction in early feeding trials was successful [16,17,66], the responsible toxins in horses have not yet been identified.

The characteristic seasonal behavior of Crofton weed pneumotoxicity in horses has led to the consideration of two major non-mutually exclusive hypotheses to explain the higher toxicity of flowering plants: (1) a higher concentration of toxins in the leaves during flowering, and (2) the possible effect of pollen as a mechanical irritant during the flowering season. The first hypothesis is supported by the typical accumulation of specific phytochemicals in the foliage of several plants during or around the flowering period [90,91,92]. Specifically, the concentration of allelochemicals and defensive compounds in invasive plants varies across different reproductive stages and parts of the plant [55,93]. The pollen of various plants in the genus Ageratina typically contains toxic compounds such as pyrrolizidine alkaloids [65,87,93]. Therefore, an increased concentration of phytotoxins in the leaves and pollen might contribute to toxicity if the whole flowering plant is ingested, or if there is a substantial amount of pollen accumulated on the leaves. A comparative phytochemical analysis of different parts of the plant possibly involved in animal toxicity (e.g., leaves, flowers, and pollen) during flowering and non-flowering periods would be informative.

As it is only possible to investigate the effects of toxins on a limited number of animal species and exposure scenarios (Table 1), translating the research conducted in model species into spontaneous disease in horses is challenging. Reproducing pneumotoxicity in other animal models has been unsuccessful, as Crofton weed induces organ-selective toxicoses across different animal species [16,17,24,25,27,28,29,44,51,69,70,71,72,73,74,75,76,77]. The role of terpenes in Crofton weed pneumotoxicity in horses has not been investigated; however, the identification of phytotoxins, including terpenes and alkaloids, in blood is possible and might be a suitable screening method for future research in cases where plant toxicity is suspected [94]. Additionally, the role of the intestinal microbiome of horses and the potential for microbial biotransformation of plant compounds into toxins have not been investigated. Microbial biotransformation has been identified as having a role in other plant toxicities, and it is noted that the only other species that presents with pulmonary abnormalities after the ingestion of Crofton weed is the rabbit, which is another hind-gut fermenter (Table 1).

Crofton weed toxicity in horses displays similar clinicopathological findings to *Crotalariosis equorum* “Jaagsiekte” [47], a respiratory disease of horses in South Africa and Northern Australia following the ingestion of *Crotarlaria* spp. Similar to equine Crofton weed pneumotoxicity, *Crotalariosis equorum* is characterized by interstitial pneumonia and the proliferation of pulmonary Clara cells, suggesting intoxication by pyrrolizidine alkaloids, possibly bioactivated via the cytochrome P450 monooxygenase system by Clara cells located in the terminal bronchioles [78]. Our current understanding of the cytochrome P450-related biotransformation processes in horses is still incomplete. However, important differences in the concentration of biotransformative enzymes in the liver and lungs of horses and other animal species in response to different drugs and xenobiotics have been identified [82,83,84,85,86]. Though the exact bioactivation mechanisms of pyrrolizidine alkaloids and sesquiterpenoids in horses have not been characterized, a predominant cytochrome P450-mediated pulmonary bioactivation of phytotoxins bypassing hepatic metabolism is possible. Indeed, besides horses, pulmonary-selective toxicities mediated through pulmonary P450 bioactivation after toxicant oral ingestion have been documented in several animal species [95,96,97]. Previous works in the literature have demonstrated the potential of in vitro models (e.g., lung microsomes, pneumocyte type II, and hepatocyte cultured cells) to investigate the role of pulmonary and hepatic biotransformative enzymes after exposure to lung-specific toxicants [98,99]. Indeed, mechanistic studies using equine-derived in vitro models could provide new information on the mechanisms of equine Crofton weed pneumotoxicity.

Crofton weed induces interstitial pneumonia in horses, characterized by multinodular pulmonary fibrosis. Though uncommon, this multinodular pulmonary fibrosis and interstitial pneumonia in horses is histologically distinctive and might be induced by only a handful of infectious agents and toxins [19,20,21,22,23]. The most morphologically similar being is Equine Herpesvirus 5 (EHV 5) [21,100], which was only discovered in the late 2000s, and is a possible differential diagnosis for Crofton weed intoxication. The complex pathogenesis and severe pulmonary fibrosis associated with the interstitial pneumonia caused by Crofton weed have limited the development of therapeutic strategies [19,20,21,22,23]. Further studies investigating the complex molecular mechanisms behind pulmonary fibrosis in horses are necessary to identify specific targets mediating tissue remodeling, such as fibroblast activation, the epithelial–mesenchymal transition, and the excessive accumulation of an extracellular matrix. Interestingly, feral herbivores and migratory goats sporadically consuming the plant do not develop toxicity leading to the postulation of possible mechanisms behind toxicity resistance [14,30,38,101]. Beneficial ruminal bacteria isolated from animals exhibiting resistance to Crofton weed toxicity might play a role, as possible prophylactic or therapeutic strategy [7].

## 5. Conclusions

This review has integrated cross-disciplinary research findings to identify the key areas for future research: (1) to identify the specific toxin/s causing pneumotoxicity in horses; (2) to establish the palatability, toxic dose, disease progression parameters, prevalence, and geographic distribution of Crofton weed cases; (3) to identify the suitable screening methods for the detection of suspected toxins in affected animals’ blood and tissues; (4) to identify the possible phytotoxins across different parts and growth stages of the plant; (5) to characterize relevant mechanistic processes and biotransformative enzyme concentrations using in vitro models and equine tissue cultures; and (6) to investigate the potential prophylactic and therapeutic strategies to ameliorate the impacts of toxicity in horses, including the use of intestinal bacteria that are capable of degrading toxins.

Crofton weed and Mist weed are easily recognizable, highly invasive global weeds. In geographic regions where Crofton weed and Mist weed are established, education of veterinarians and their clients about the risks posed, how to recognize the plants in situ, and control measures is critical [14]. Once the plants are identified, horse owners must prevent any grazing of infiltrated pastures [31,32]. Ingestion results in pulmonary fibrosis, which is ultimately fatal. Horses present with exercise intolerance, increased respiratory rates, and progressive dyspnea. Although not well documented, pulmonary changes should be identifiable ultrasonographically and radiographically. Fibrosis observed on histopathology of lung samples collected antemortem by percutaneous biopsy or at necropsy without the identification of etiologic agents such as fungal hyphae or EHV5 by PCR should make Crofton weed pneumotoxicity a likely differential in horses grazing in paddocks with access to Crofton weed or Mist weed.

## Figures and Tables

**Table 1:** Toxicity-related findings after experimental exposure to Crofton weed, plant extracts, and purified toxins in several animal species.

Species	Fed as	Administration	Identified or Administered Toxin	Toxicity Notes
Horses	Whole plant [11,12]	PO [11,12], IG [12]	NI. [11,12]	Pneumotoxicity [11,12]
Rabbits	Whole plant [11]	PO [11]	NI. [11]	Pulmonary histopathological abnormalities [11]
Sheep	Whole plant [11]	PO and IG [11]	NI [11]	No toxicity observed [11]
Cattle	Leaves [50]	PO [50]	NI [50]	Anorexia, rumen suspension, and photosensitisation [50]
Goats	Fresh leaves [51] and dried leaves [21,22,23]	PO [21,22,23,51]	NI [21,22,23,51]	Renal toxicity [21], spleen toxicity [22], hepatotoxicity [23], and inappetence [51]
Mice	Leaf powder [52], pelleted plant [20], freeze-dried LP [53], ME [19,54], and purified toxins [55,56]	PO [19,52,53,54,55], IG [56,57]	Euptox A [19,55,56], DAOA [55], and OA [55]	Hepatotoxicity [19,52,53,54,55,57] and spleen toxicity [20,55,56]
Rats	Whole plant [11], freeze-dried LP [18], air-dried LP [58], oven-dried leaves [46,59], feed containing ME [59], and purified toxins [60]	PO [11,18,46,58,59,60]	Euptox A [60]	No toxicity observed [11], hepatotoxicity [18,46,59,60], intestinal damage, and intestinal immune barrier dysfunction [58]

Routes of administration: LP, leaf powder; ME, methanol extract or food containing ME; PO, per os (spontaneous oral consumption or oral administration); IG, intra gastric (feeding tube). Toxin: NI, not identified; Euptox A, 9-oxo-10, 11-dehydroagerophorone; DAOA, 2-deoxo-2-(acetyloxy)-9-oxo-ageraphorone; and OA, 9-oxoagerophorone.
